# Polyamine catabolism adds fuel to leaf senescence

**DOI:** 10.1007/s00726-016-2377-y

**Published:** 2016-12-30

**Authors:** E. Sobieszczuk-Nowicka

**Affiliations:** Department of Plant Physiology, Faculty of Biology, Adam Mickiewicz University of Poznań, ul. Umultowska 89, 61-614 Poznań, Poland

**Keywords:** Barley, Leaf, Polyamines, Catabolism, Senescence, Transglutaminases

## Abstract

Leaf senescence is a terminal step in plant growth and development. Considerable information on processes and signals involved in this process has been obtained, although comparatively little is known about leaf senescence in monocotyledonous plants. In particular, little is known about players involved in leaf senescence imposed by a prolonged dark treatment. New information has now been unveiled on dark-induced leaf senescence in a monocot, barley. A close association has been found between ubiquitous polyamines, reactive oxygen species (ROS), and senescence of barley leaves during prolonged darkness. Although polyamines (putrescine, spermidine, and spermine) are absolutely essential for critical cellular functions, including regulation of nucleic acids and protein synthesis, macromolecular structural integrity, and signalling, a strong link between polyamines and dark-induced leaf senescence has been found using barley plant as a model of monocots. Interestingly, *Arabidopsis* polyamine back-conversion oxidase mutants deficient in the conversion of spermine to spermidine and/or spermidine to putrescine do not occur and have delayed entry into dark-induced leaf senescence. This review summarizes the recent molecular, physiological, and biochemical evidence implicating concurrently polyamines and ethylene in dark-induced leaf senescence and broadening our knowledge on the mechanistic events involved in this important plant death process.

## Introduction

The phenomenon of senescence is a ubiquitous characteristic of the biological world. From an ontogenetic perspective, biologists consider senescence as an evolutionarily acquired and genetically programmed developmental process. The most prominently studied senescence process in plants is leaf senescence (Woo et al. 2013). Regulation of leaf senescence involves multiple layers of control, including hormonal cues among which ethylene features prominently. Numerous studies have also linked polyamines (PAs) to the regulation of plant cell senescence. The main PAs in plants include putrescine (Put), spermidine (Spd), spermine (Spm), and thermo-Spm (t-Spm). PAs have been implicated in the prolonged survival of excised organs or senescing organs in vivo, namely, leaves, flowers, and fruits (reviewed in Cai et al. 2015). However, there are contradictions about whether PA levels increase or decrease during senescence (Cai et al. 2015). Many studies have focused on the involvement of PA in plant senescence, using exogenous application of pharmacological doses or by genetic means to overproduce PAs (Cohen et al. 1979; Mizrahi et al. 1989; Besford et al. 1993; Legocka and Zajchert 1999; Mehta et al. 2002; Mattoo et al. 2006; Mattoo and Handa 2008; Nambeesan et al. 2010; Serafini-Fracassini et al. 2010). In addition, quantification of free PAs in tissue extracts has provided a “snapshot” picture of their levels during a continuously changing environment, since intra-cellular levels of PAs reflect the balance of their synthesis, catabolism, attachment to other molecules, or transport (Kusano and Suzuki 2015). It is now known that PA metabolism during senescence is linked to many intra-cellular metabolic pathways, including signalling molecules and metabolites associated with cellular response to environmental changes. In brief, the findings indicate that the internal PA pool undergoes regulation in senescing leaves. More information is becoming available on how PA metabolism is linked to physiological changes that ultimately lead to cell death and the nature of changes in the level of the free, conjugated or bound form of PAs. Processes interlinked with the increase or decrease in PA titer during senescence and the ability of plants to control senescence in relation to their capacity to metabolize PAs are slowly being unearthed (Sobieszczuk-Nowicka et al. 2015, 2016; Sequera-Mutiozabal et al. 2016). Recent studies on leaf senescence in a monocotyledonous crop plant, barley, demonstrate an important issue in relation to the crop yield and impact on sustainability of agricultural crops.

### Polyamines in leaf senescence: early history

Leaf senescence involves three phases: initiation, degradation, and termination. The initiation phase starts with changes in the gene expression profiles, particularly in genes encoding proteins for chlorophyll degradation. Leaf yellowing is one of the first visible morphological symptoms of senescence. Other important changes within the senescing leaf cells are ultra-structural modifications, including decay of the cytoskeleton, fragmentation of the endoplasmic reticulum, degradation of ribosomes, and structural changes within chloroplasts (Thomas et al. 2003).

That PAs may be important for controlling the leaf senescence process in barley became apparent when a decrease in the endogenous levels of free PAs in senescing chloroplasts was observed (reviewed in: Sobieszczuk-Nowicka and Legocka 2014). Polyamines are multi-functional, ubiquitous polycationic compounds involved in many physiological and developmental processes, as well as stress tolerance, with much research focused mostly on Put, Spd, and Spm plus a recent addition in thermo-Spm (Mattoo and Handa 2008; Takahashi and Kakehi 2010; Kusano and Suzuki 2015). The involvement of PAs in the prevention of senescence was heralded by early research in the laboratory of Arthur Galston at Yale University, studies that utilized pharmacological doses of PAs and indicated increased protoplast viability, delay in senescence, decrease in ribonuclease activity, induction of DNA synthesis, and mitosis in plant protoplasts (Galston et al. 1978; Kaur-Sawhney et al. 1978). The effects of exogenously applied PAs became a common feature thereafter (Cohen et al.^1979^; Apelbaum et al. 1981; Mizrahi et al.^1989^; Besford et al. 1993; Legocka and Zajchert 1999; Serafini-Fracassini et al. 2010). In particular, PAs were shown to delay senescence in oat and *Petunia* leaves, and PAs were found strongly bound to high-molecular weight proteins (Mizrahi et al. 1989). In oat leaves exposed to osmotic stress, the exogenously applied Put caused chlorophyll degradation and rapid senescence concomitant with Put accumulation. However, exogenous addition of Spd or Spm inhibited protein degradation, chlorophyll loss, and stabilized thylakoid proteins, such as D1, D2, cytochrome f, and large subunit of carbon fixing enzyme ribulose-bisphosphate carboxylase/oxygenase (Rubisco) enzyme (Besford et al. 1993; Legocka and Zajchert 1999). Treatment of excised leaves of barley senescing in darkness with Spd led to inhibition of RNase activity, chlorophyll degradation, and LHCII protein degradation (Legocka and Zajchert 1999), while applied Spm caused a delay in chlorophyll *b* degradation and of some plastid proteins, while an increase in Ca^2+^-dependent transglutaminases (TGases, E.C. 2.3.2.13) activity was apparent (Serafini-Fracassini et al. 2010). TGases catalyze post-translational modification of proteins by establishing covalent linkage of ε-(γ-glutamyl) moeity on PAs (Serafini-Fracassini and Del Duca 2008). Spm treatment of senescing *Lactuca* leaves in planta had similar effects and TGase activity was reactivated (Serafini-Fracassini et al. 2010).

In development-related and dark-induced *Avena sativa* L. senescing leaves, arginine decarboxylase (ADC, EC 4.1.1.19) activity decreased progressively, while ornithine decarboxylase (ODC, EC 4.1.1.17) activity was high and constant in aging leaves but decreased in those kept in the dark (Kaur-Sawhney et al. 1982). S-adenosyl-l-methionine decarboxylase (SAMDC, EC 4.1.1.50) activity was not correlated with age or senescence. Put, diaminopropane (Dap), agmatine, and Spm levels were high in young leaves and declined with age. The best single indicator of leaf senescence was Spm, which decreased in excised leaves incubated in the dark (Kaur-Sawhney et al. 1982).

### Polyamines in leaf senescence: recent studies

PA biosynthesis, catabolism, conjugation, interconversion, and transport contribute to PA homeostasis (Moschou and Roubelakis-Angelakis 2013). PA titer in plant cells is highly regulated (Cogen 1998; Moschou et al. 2008; 2012; Angelini et al. 2010; Moschou and Roubelakis-Angelakis 2013; and references therein). Transformations between individual PAs may essentially contribute to darkness-induced responses, and this was highlighted in barley leaf senescence (Sobieszczuk-Nowicka et al. 2016). The question arises: “Is the increase in free PA titer at the beginning of senescence a part of a signalling mechanism that leads the cell to its death.” PA accumulation upon senescence is linked to up-regulation of PA biosynthesis gene and consequently to increase in the corresponding enzymatic activities. Senescence is sensitive to hormonal perturbation, particularly to cytokinins (Lim et al. 2007). Kinetin (KIN) treatment delays PA accumulation and it has been implied that some senescence-related signals blocked by KIN influence PA synthesis (Sobieszczuk-Nowicka et al. 2016). It remains to be established if PA accumulation induced at the beginning of the senescence process has any reactive oxygen species-scavenging (ROS) function (Radyukina et al. 2009; Legocka et al. 2015).

Transcript levels and corresponding activation of PA catabolic enzymes, DAO and PAO, increase during dark-induced senescence, and are therefore considered important components of senescence-related mechanisms (Ioannidis et al. 2014; Sobieszczuk-Nowicka et al. 2016). Inhibiting PAO activity drastically slowed down the accumulation of both Dap and Put, while the levels of Spd and Spm were substantially increased. This is expected but, remarkably, this also resulted in slowing down the senescence-associated chlorophyll loss. Furthermore, inhibition of PAO activity led to decreased H_2_O_2_ levels, suggesting that PAO-mediated catabolism of Spd and/or Spm supports dark-induced senescence. In this regard, *Arabidopsis* PA back-conversion oxidase mutants, in which conversion of Spm to Spd and/or Spd to Put does not occur, have delayed entry into dark-induced senescence (Sequera-Mutiozabal et al. 2016). Delayed dark-induced senescence in mutants is associated with higher Spm level and lower Spd/Spm ratio (Sequera-Mutiozabal et al. 2016). *AtPAO4*, a member of the five *Arabidopsis PAO* gene family, has high affinity for Spm oxidation, transforming Spm into Spd via back-conversion, but not Spd into Put (Takahashi et al. 2004; Kamada-Nobusada et al. 2008; Fincato et al. 2012). Delayed leaf senescence is associated with higher Spm level, reduced ROS production, and increased nitric oxide (NO) levels. A synthesis of these data suggests that Spm is a ‘signalling’ metabolite, providing protection against stress through metabolic conversions that involve ascorbate/dehydro-ascorbate redox state modifications, changes in sugar and nitrogen metabolism, cross-talk with ethylene biosynthesis, and mitochondrial electron transport chain modulation (Sequera-Mutiozabal et al. 2016). Thus, metabolic interactions between PAs, particularly Spm, occur with cell oxidative balance and transport/biosynthesis of amino acids, likely a strategy to cope with oxidative damage during senescence.

Plant responses to environmental factors involve the secretion of Spd to the apoplast, where their catabolism leads to H_2_O_2_ production as is known in hypersensitive plant response. Dependent upon the amount of H_2_O_2_, the defence response or cell death program is initiated (Yoda et al. 2003, 2006, Marina et al. 2008; Moschou et al. 2008). Interestingly, in this regard, high Sdp and Spm pool were found in the apoplast during dark-induced leaf senescence. This resulted in gradual accumulation of apoplastic Dap and H_2_O_2_ (Sobieszczuk-Nowicka et al. 2016). The initial amount of Put in the apoplastic pool of PAs is one order of magnitude lower and increases only slightly during senescence. However, Put dominates in the free PA fraction, initially accumulating to high levels before decreasing. It is noted here that the decrease in free Put is accompanied by the formation of Put conjugates that accumulate in the senescing leaf to high levels, indicating that the Put-conjugating enzymes are active in the senescing cell (Sobieszczuk-Nowicka et al. 2016). Senescence-dependent remobilized nitrogen (N) and carbon (C) flow may contribute to PA conjugation, since the expression of respective protein coding genes also increases (Sobieszczuk-Nowicka et al. 2016). That PAs are sensed by plant cells as organic-N and stimulate turnover of N molecules has been previously discussed (Mattoo et al. 2006, 2010).

Another interesting facet is the involvement of a DAO-mediated Put oxidation process in γ-aminobutyric acid (GABA) production. Microarray-based profiling of glutamate decarboxylase gene expression suggested that in dark-induced senescing leaves GABA synthesis from glutamate is gradually suppressed (Sobieszczuk-Nowicka et al. 2016). Put oxidation could contribute to the alternative source of GABA and possibly other signalling pathways. Blocking Put oxidation pathway accelerated chlorophyll degradation, as was demonstrated from *Chl a* fluorescence parameters and plant N status. Important to note is that simultaneous addition of exogenous GABA together with a DAO inhibitor is sufficient to prevent accelerated degradation of chloroplast photosystem complexes (Sobieszczuk-Nowicka et al. 2016). Together, these results highlight a central role for GABA signalling in senescing organ and favour the conclusion that Put can act as a key precursor of this neurotransmitter.

Hormonal regulation of plant senescence involves the hormone ethylene (Fluhr and Mattoo 1996; Woo et al. 2013). In this regard, PAs seem to be anti-senescence regulators by inhibiting ethylene, and, conversely, ethylene inhibits the biosynthesis of polyamines (Fluhr and Mattoo 1996; Cassol and Mattoo 2003; Nambeesan et al. 2010; Harpaz-Saad et al. 2012; Anwar et al. 2015). A possibility of temporal relationship between PAs and ethylene during plant development has been previously presented, wherein competition for SAM, which is an early precursor for both PAs and ethylene, has been discussed (Fluhr and Mattoo 1996; Cassol and Mattoo 2003; Harpaz-Saad et al. 2012). In addition, the fact that biosynthesis of PAs and ethylene can co-exist simultaneously first presented with studies on tomato fruit (Mehta et al. 2002) was corroborated in dark-induced leaf senescence phenomenon in barley (Sobieszczuk-Nowicka et al. 2016).

Physiological and structural changes in chloroplasts of barley leaves during dark-induced senescence are associated with PA conjugation, modification of chloroplast proteins, and modulation of chloroplast-localized transglutaminases (ChlTGases). Thus, in situ localization and changes in the ChlTGase activity during dark-induced senescence mirror increase in the level of plastid membrane-bound Put and Spd (Sobieszczuk-Nowicka et al. 2009, 2015).

ChlTGase catalyzes binding of [^3^H]Put and [^3^H]Spd to photosystem proteins (Sobieszczuk-Nowicka et al. 2015). Substrates of ChlTGases in mature leaves include apoproteins of the chlorophyll a/b antenna complex, LHCII, ATP synthase, and pSbS (photosystem II 22 kDa protein), known proteins that are essential in energy-dependent quenching and increased thermal dissipation of excessively absorbed light energy in the photosystems (Del Duca et al. 1994; Dondini et al. 2003; Della Mea et al. 2004a; Campos et al. 2010). Several stress-responsive proteins detected in the polyamine-bound fraction only after dark-induced senescence include the antioxidant enzyme peroxiredoxin, heat shock protein, ent-copalyldiphosphate synthase, and IAA-amino acid hydrolase (Wang et al. 2004; Van der Graaff et al. 2006; Noushina et al. 2011; Cejudo et al. 2012). That PAs in concert with TGases are functionally involved in the dark-induced leaf senescence that is supported by proteomic analysis and TGase activity/transcript modulation (Sobieszczuk-Nowicka et al. 2009, 2015). The most studied plant gene coding for a protein with TGase activity is *Arabidopsis AtPng1p*. *AtPng1p* is constitutively expressed at low levels in all plant organs during various stages of development and under various light conditions (Della Mea et al. 2004b). Similar expression pattern was found for the *HvPng1*-*like* homolog in barley. However, *HvPng1*-*like* transcripts actually increased as soon as senescence was induced in the dark being concomitant with cell structure disintegration initiation (Sobieszczuk-Nowicka et al. 2015).

**Fig. 1:**
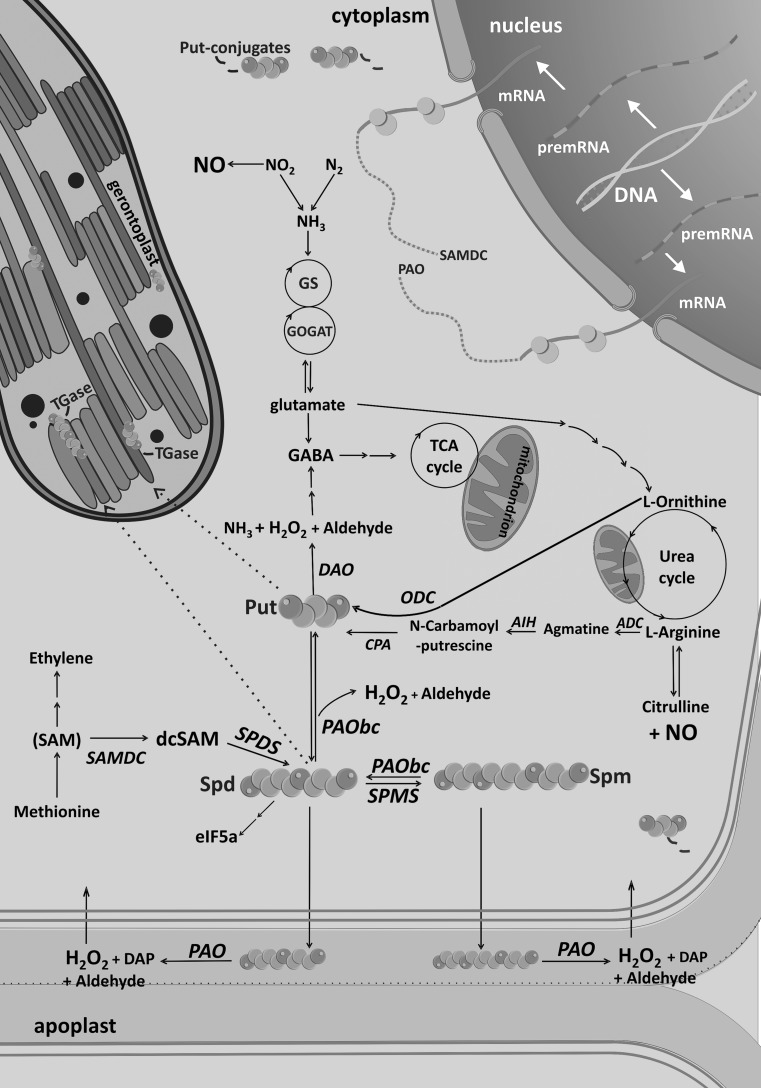
Polyamines and dark-induced barley leaf senescence. Polyamine (PA) metabolism is linked to many metabolic pathways in the cell among others by being involved in the formation of signalling molecules and metabolites directly related to the cellular response to senescence, namely, ethylene, γ-aminobutyric acid (GABA), tricarboxylic acid cycle (TCA) metabolites, urea cycle metabolites, amino acids (glutamine, glutamate), arginine, ornithine, hydrogen peroxide (H_2_O_2_), nitric oxide (NO), and translation factor (eIF5a precursor). At the beginning of the dark-induced senescence process, a rapid increase in the level of free putrescine (Put), spermidine (Spd), and spermine (Spm) is observed, due likely to simultaneous up-regulation of a set of genes involved in PA biosynthesis and an increase in enzymatic activity of the proteins they encode. This effect is accompanied by the formation of Put conjugates that accumulate to high levels in the senescing leaf. Senescence-dependent nitrogen and carbon flow might be shifted toward PA conjugation. At the later stages of the process, the levels of PAs begin to drop and are preceded by an increase in transcript levels and activity of the PA catabolic enzymes. Diamine oxidase-mediated Put oxidation is GABA production. Put oxidation is an alternative source of GABA to TCA and possibly for some signalling pathways. Furthermore, PA catabolism through senescence is expressed as Spd and Spm production and their transport into the apoplast, where they produce H_2_O_2_ and diaminopropane (Dap), both of which can participate in senescence-dependent degradation processes. Dark-induced leaf senescence also corresponds to a wide contribution of PAs to dark-induced senescence-associated responses within chloroplast, where PAs can be transported or synthesized *de novo*. Identification of post-translational modification of plastid proteins by PAs (PA-conjugated proteins) via transglutaminases (TGases) during senescence suggests that PAs contribute to senescence-related stress response, inhibition of photosynthesis and cell death, chloroplast-to-gerontoplast conversion, and cellular disintegration. *ADC* arginine decarboxylase, *AIH* agmatine iminohydrolase, *CPA* N-carbamoylputrescine amidohydrolase, *ODC* ornithine decarboxylase, *SAM* S-adenosylmethionine, *SAMS* SAM synthetase, *SAMDC* SAM decarboxylase, *SPDS* spermidine synthase, *SPMS* spermine synthase, *PAO* polyamine oxidase, *PAObc* back-conversion polyamine oxidase, *DAO* diamine oxidase

The knowledge of the participation of PAs in leaf senescence is still very fragmentary. Results reported here shed some light on the problem, in particular on leaf senescence in an important crop. These insights allow the development of a frame work that would provide more detailed observations into induced senescence and its biotechnological applications, and would stimulate new, important questions about the function of PAs in the process. A model depicting PAs function in dark-induced senescence is summarized in Fig. 1.

## Conclusions

The development of high yielding and nutritious crops has become a central challenge of this century. The impact of induced senescence provides a window on senescence-related crop yield and quality. The demand is to limit pre- and post-harvest losses which are estimated to be close to or >30%. Significant advances have been made in our understanding of leaf senescence syndrome and its underlying regulation (Schipper et al. 2015 and references therein). In addition, a theoretical model (senescence window concept, Jing et al. 2002) has emerged with a scenario of how the capacity to senescence is established during leaf development and how internal and external factors are integrated with age to define the timing of senescence. It is extremely difficult to uncouple senescence regulatory pathways from stress responses, since the genetic program(s) underlying senescence largely overlaps with that of plant defence (reviewed in Schipper et al. 2015). Therefore, altering one senescence parameter might also reduce the plant tolerance to stress.

Metabolism of PAs, mainly the catabolism of Spd and Spm by PAOs, has been proposed to promote senescence and PCD in leaves through production of H_2_O_2_ and ensuing oxidative stress. Senescence initiated in leaves due to long duration in the dark modulate PA levels through changes in gene expression as well as the activities of the corresponding enzymes involved in PA metabolism (Sobieszczuk-Nowicka et al. 2016) (Fig. 1). Clearly, PAs and plant senescence cross the paths (Del Duca et al. 2014 and references therein; Ioannidis et al. 2014; Cai et al. 2015 and references therein; Sequera-Mutiozabal et al. 2016; Sobieszczuk-Nowicka et al. 2015, 2016). However, conclusive evidence in favour of this link in normal plant senescence has yet to be established. Physiological functions of PAs in senescence need to be gradually clarified at the molecular level. Studies using *Escherichia coli* strongly suggest that polyamine effect on cell viability mainly occurs at the level of translation through interaction with RNA (Igarashi and Kashiwagi 2010). In animals, it was recently found that four kinds of genes are members of the polyamine modulon: genes encoding proteins, whose synthesis is enhanced by PAs at the level of translation (reviewed in Igarashi et al. 2015). In addition, eukaryotic initiation factor 5A (eIF5A) contains hypusine, which is a modified lysine with the addition of the 4-aminobutyl moeity from Spd, that is known to regulate protein translation (Park et al. 2010). These findings support the idea that in plant senescence, PAs may regulate the process through regulation of protein synthesis.

Finally, SAM is a precursor for the biosynthetic pathways of both PAs and the leaf senescence promoter ethylene. Being a major substrate in 1-C metabolism and involved in methylation processes, SAM diverted to PAs and/or ethylene during leaf senescence needs to be assessed metabolically, as has been described recently in the tomato system (Lasanajak et al. 2014). Regulation of SAM levels in mammalian cells was found regulated involving post-translational inhibition of glycine *N*-methyltransferase by folate (Luka et al., 2009). How plant cells regulate SAM levels during plant senescence is an important avenue to explore.

## Abbreviations

PAs: Polyamines
PCD: Programmed cell death
Put: Putrescine
Spd: Spermidine
Spm: Spermine
TGase: Transglutaminase
